# Fractures

**Published:** 1901-06

**Authors:** 


**Fractures.** By Carl Beck, M.D. Pp. 335. Philadelphia: W. B. Saunders & Co. 1900.

In the preface the author says the use of the Röntgen ray "has led to many and revolutionising discoveries; most of these have marked a clearer understanding, and consequently the better treatment of fractures. . . . This book is an effort to encompass, in a systematic treatise, the important essentials of the publications on this subject."

All the fractures in the different regions of the body are taken seriatim, and the symptoms and treatment appropriate to each are discussed; and, in addition to this, all the common forms of fracture, and many of the rarer forms, are skiagraphically illustrated. These skiagrams are for the most part excellent reproductions, but some of those of the deeper parts of the body and of the skull are not particularly clear, and do not much help the elucidation of the text.

As an example of how much skiagraphy has done to make our knowledge of fractures more exact, take the fractures of the lower end of the humerus. The author divides these into four kinds: supracondylar, diacondylar, epicondylar, and intercondylar, and this seems to be a very good way of classifying these fractures. Again, take the fractures of the lower end of the radius massed together under the name of Colles' fracture. These are here classified into—epiphyseal separation, fissures (infractions), complete fractures, incomplete fractures, fractures of the lower end of the radius combined with infraction or fracture of the head of the ulna, and fractures of the lower end of the radius combined with fractures of the styloid process of the ulna. (This latter complication the author finds to be present in 32 per cent. of all cases of fracture of the lower end of the radius.)

The chapter on general treatment at the beginning of the book is exceedingly practical and full of sound common sense, and the author is not at all afraid of expressing his opinion; *e.g.*, he dismisses the question of wiring the fragments in a simple subcutaneous fracture by calling it a "surgical aberration" (p. 51). Again, when discussing the wiring of the fragments in fractured patella, he says: "Complicated manœuvres, like boring holes into the fragments, &c., cannot be too strongly condemned, since simply conducting a large needle armed with silver wire around the fragments secures their perfect opposition" (p. 201). This statement a good many surgeons would take exception to.

Not the least useful part of the book is the chapter on "Errors of Skiagraphy," pointing out the difficulties of reading a skiagram correctly, and the danger of relying for a diagnosis on a single skiagram, it being absolutely necessary in many cases to have skiagrams taken from more than one point of view.

The book on the whole is distinctly a useful clinical treatise on fractures, and shows well how much our knowledge of fractures has been increased by the use of the X-rays.

